# Solubility and Diffusion of Main Biogas Components in a Glassy Polysulfone-Based Membrane

**DOI:** 10.3390/molecules30030614

**Published:** 2025-01-30

**Authors:** Marek Tańczyk, Aleksandra Janusz-Cygan, Anna Pawlaczyk-Kurek, Łukasz Hamryszak, Jolanta Jaschik, Katarzyna Janusz-Szymańska

**Affiliations:** 1 Institute of Chemical Engineering, Polish Academy of Sciences, Bałtycka 5, 44-100 Gliwice, Poland; ajcygan@iich.gliwice.pl (A.J.-C.); ania.pawlaczyk@iich.gliwice.pl (A.P.-K.); lukasz.hamryszak@iich.gliwice.pl (Ł.H.); jjaschik@iich.gliwice.pl (J.J.); 2 Department of Power Engineering and Turbomachinery, Faculty of Energy and Environmental Engineering, Silesian University of Technology, Konarskiego 18, 44-100 Gliwice, Poland; katarzyna.janusz-szymanska@polsl.pl

**Keywords:** biogas separation, polysulfone-based membrane, competitive sorption, glassy polymer, Dual Mode Sorption model, partial immobilization model, hybrid processes, membrane swelling

## Abstract

Biogas, one of the important controllable renewable energy sources, may be split into two streams: bio-CH_4_ and bio-CO_2_ using, among others, membrane processes. The proper optimization of such processes requires the knowledge of phenomena accompanying each specific CH_4_–CO_2_–membrane system (e.g., competitive sorption or swelling). The phenomena were analyzed for the polysulfone-based membrane used in a developed adsorptive–membrane system for biogas separation. The Dual Mode Sorption and partial immobilization models were used to describe the solubility and diffusion of CO_2_, CH_4_ and their mixtures in this material. The parameters of the models were determined based on pure-gas sorption isotherms measured gravimetrically and permeances of CO_2_/CH_4_ mixture components from our previous studies. It was found, among other things, that the membrane swelling caused by CO_2_ was observed for pressures higher than 5 bar. The real selectivity (permselectivity) of CO_2_ vs. CH_4_ is significantly lower than the selectivity of pure gases (ideal selectivity), while the solubility selectivity of CO_2_ vs. CH_4_ in the mixture is higher than that of pure gases. This is due to the better affinity of CO_2_ towards the tested polysulfone membrane, making CO_2_ the dominant component in competitive sorption. The reduction in the permselectivity is mainly due to an approximately two-fold decrease in the CO_2_ diffusion rate in the presence of CH_4_. It was also found that the fraction of solubility in the fractional free volume (FFV) is dominant for both gases, pure and mixed, reaching 65–73% of the total solubility. Moreover, in CO_2_/CH_4_ mixtures, the mobility of methane in FFV disappears, which additionally confirms the displacement of methane by CO_2_ from FFV.

## 1. Introduction

**Figure 1:**
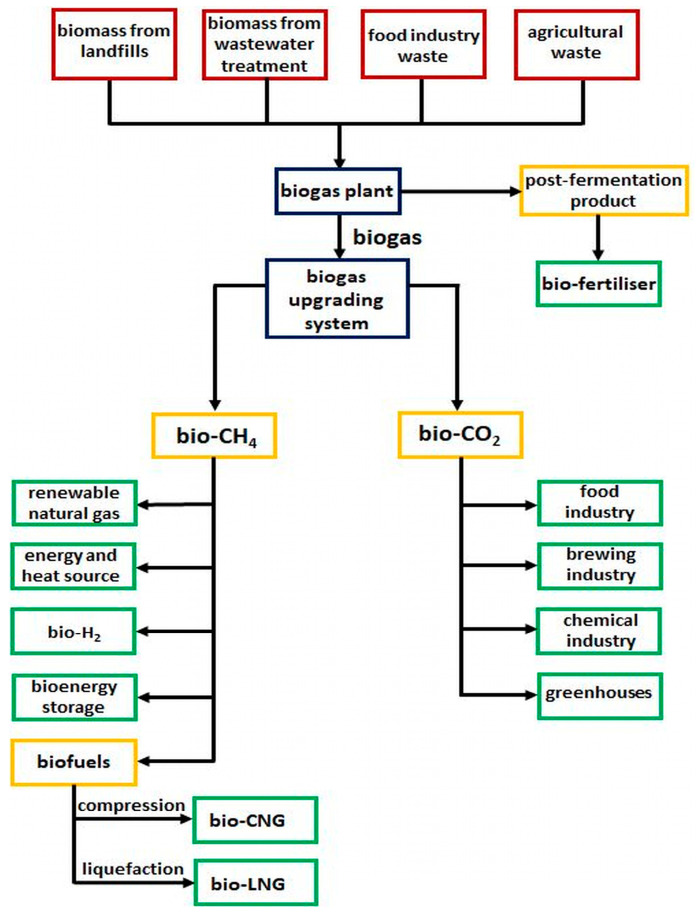
Biomethane value chain.

The global geopolitical situation, the European Green Deal and the associated Methane Emissions Reduction Strategy are forcing Europe to transition from fossil fuels to green energy sources. However, using the wind or sun is related to constant fluctuations due to changes in weather or time of the day. With the increasing participation of wind and solar energy, the flexibility of the energy system, which must react quickly to fluctuations in supply and demand as well as maintain the grid stability, becomes increasingly important. This can be achieved, among other things, by the development of biogas-based energy, as biogas is one of the so-called controllable RES (renewable energy sources). Most of the biogas currently produced in Europe is burned in cogeneration units to produce electricity and heat. Due to the fact that biogas plants are built at a distance from the inhabited area, the degree of usage of the produced heat is not satisfactory. An alternate option is to upgrade biogas to biomethane. Biogas is a gas mixture that is a product of the methane fermentation of organic compounds, consisting mainly of methane (45–70% by volume) and carbon dioxide (25–45% by volume) as well as trace amounts of other gases (hydrogen sulfide, nitrogen, oxygen, siloxanes) [1,2,3], while biomethane is a refined biogas with parameters of natural gas. The benefits that can be achieved by converting biogas to biomethane are shown in Figure 1.

Technologies for biogas–to–biomethane upgrading have already found practical applications on a large scale (several hundred m^3^ h^−1^ biogas) [4,5,6,7]. According to the European Biogas Association (EBA), 4.2 billion m^3^ of biomethane were produced in Europe in 2022, and the goal is to produce 35 billion m^3^ of biomethane in 2030 [8]. At the end of 2022, there were 1323 biomethane installations in Europe [9], and the amount is steadily increasing. Despite the success that membrane technologies have achieved on an industrial scale, intensive research and development are still underway, which focus both on the manufacture of innovative membrane materials with specific separation properties and the finding of new process solutions that would enable the separation of biogas into two useful streams: bio-CH_4_ and bio-CO_2_.

Biogas can be split into these two streams using, among other things, technologies developed for CO_2_ separation from flue gases, such as a hybrid VSA–membrane installation [10]. In this installation, the second membrane stage, based on commercially available polysulfone- and polyimide-based membranes, produces CO_2_-rich gas. This second stage is fed with a gaseous mixture containing 50–70 vol.% of carbon dioxide at a pressure of 3–10 bar and should be optimized for CO_2_/CH_4_ separation.

In simulations of the membrane process for biogas separation, it is necessary to take into account the multicomponent nature of the mass transport through the membrane in the model [3,11,12,13,14]. Of course, one can theoretically anticipate the permeation properties of gas mixtures based on the permeances determined for pure gases. In fact, the membrane selectivity thus determined (called ideal selectivity, see Equation (2)) is often overestimated, due to phenomena such as competitive sorption, plasticization, swelling or the non-ideality of gases [15]. This is also the case for the CH_4_–CO_2_–membrane system, where competitive sorption occurs in the voids of the glassy polymer [12,13], among other things, and the appropriate data are lacking for the membrane materials used in the hybrid installation. It is also well known that in many membrane materials, CO_2_-accelerated plasticization takes place [16,17,18]. In the case of CO_2_/CH_4_ mixtures, combinations of competitive sorption and plasticization are reported the most often as a reason for deviation from the ideal pure-gas selectivity described in the literature [18,19,20].

Hence, the sorption of carbon dioxide and methane in the polysulfone-based membrane from Air Products’ PRISM PA1020–P1 module was investigated experimentally in the pressure and temperature range used in the hybrid system. Moreover, the Dual Mode Sorption (DMS) model and the partial immobilization model [14] were used to describe the sorption and transport properties of CO_2_/CH_4_ mixtures in the studied glassy polymer membrane. The models enable the identification and explanation of the solubility and diffusion phenomena accompanying the permeation of the main biogas components in a glassy polymer membrane. Since the DMS model takes into account competitive sorption but not the other phenomena, rigorous analysis was limited to cases where the plasticization, swelling or the non-ideality of gases could be avoided.

The analysis concerning the predictions of the DMS and partial immobilization models for CO_2_/CH_4_ mixtures was related to the experimental studies of their permeation through the Air Products’ PRISM PA1020–P1 polysulfone-based membrane module, carried out with binary mixtures containing 40 and 50 vol.% of CO_2_ [3,21]. As a result of this analysis, a set of parameters for these models was obtained, which may be used to predict CO_2_ and CH_4_ permeances required in the modeling and optimization of the membrane stage in the hybrid adsorptive–membrane process for biogas separation.

## 2. Results and Discussion

The mass transfer (permeation) of the gas compound i-th in dense polymeric membranes depends on its diffusion and solubility in the polymer structure. This is usually described by the solution–diffusion model [22] where the mass transfer coefficient, permeability (P_i_), is the product of the solubility (S_i_) and the diffusion coefficient (D_i_):

(1) $$P_{i}=S_{i}\cdot D_{i}=Q_{i}\cdot \delta_{M}$$

In the above equation, the permeability is also related to the permeance (Qi) and membrane thickness (δ_M_). The former can be directly derived from the mass flux and pressure difference on both sides of the membrane, measured in permeation experiments [3,21].

The ratio between the permeability coefficients of two gases, i-th and j-th, gives the permselectivity (α_ij_), which is the basic parameter describing the separation performance of a membrane material. In the case of carbon dioxide and methane, and taking into account the solution–diffusion model, the permselectivity of CO_2_ vs. CH_4_ may be expressed as a product of solubility selectivity (α_(S)_) and diffusivity selectivity (α_(D)_):

(2) $$\propto_{{CO}_{2}/{CH}_{4}}=\frac{P_{{CO}_{2}}}{P_{{CH}_{4}}}=\left(\frac{S_{{CO}_{2}}}{S_{{CH}_{4}}}\right)\cdot \left(\frac{D_{{CO}_{2}}}{D_{{CH}_{4}}}\right)=\propto_{{(S)CO}_{2}/{CH}_{4}}\cdot \propto_{{(D)CO}_{2}/{CH}_{4}}$$

If the parameters in Equation (2) refer to pure gases, then one speaks of ideal selectivity.

The permeances of CO_2_ and CH_4_, pure and mixed, in the PRISM PA1020–P1 Air Products module with a polysulfone-based membrane, were determined in our previous studies [3,21]. They are collected and presented for reference in Tables S1 and S3 in the Supplementary Material and used further in the discussion concerning the solubility and diffusion of carbon dioxide and methane, pure and mixed, in this membrane.

### 2.1. Solubility of Pure CO_2_, CH_4_ and Their Mixtures

The solubility of gaseous species i-th in Equations (1) and (2) is expressed as follows:

(3) $$S_{i}=\frac{C_{i}}{p_{i}}$$

where C is the gas concentration in a polymer (cm^3^(STP) cm^−3^) and p is the gas pressure (bar). The concentrations of pure carbon dioxide and methane in the polysulfone-based membrane from the Air Products PRISM PA1020 module were measured gravimetrically in the temperature range of 283–303 K. The pressure range was 0–10 and 0–5 bar in the case of methane and carbon dioxide, respectively. Additionally, the CO_2_ isotherm at 293 K was also measured for pressures from 0 to 10 bar. Each isotherm was determined in two runs: sorption and desorption. In the first one, the pressure was increased step by step from 0 to the maximum, and, in the second, the pressure was decreased the same way from the maximum pressure to 0. The experimental methodology is described in Section 3.2.

**Figure 2:**
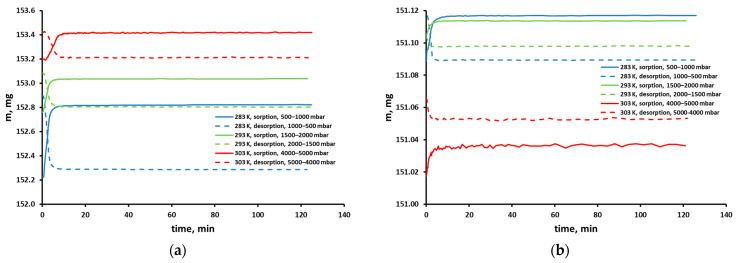
Sample mass uptake curves of pure (**a**) CO_2_ and (**b**) CH_4_ in the polysulfone-based membrane from Air Products’ PRISM PA1020–P1 module.

In Figure 2, exemplary mass uptake curves are shown for different pressures and temperatures. Such uptake curves show the change in the sample mass over time due to gas sorption related to the change in its pressure and are registered with every isotherm point. As can be seen in these figures, the full mass uptake was reached after about 10 min. The sample weight did not change after this time, which means that sorption equilibrium was achieved. The changes in the sample weight are relatively small and amount to 200–600 µg and 10–25 µg for carbon dioxide and methane, respectively. However, they significantly exceed the measurement resolution of the microbalance (0.2 μg).

The fluctuations that are clearly visible in the case of the methane sorption curve shown in Figure 2b (at 303 K and with an increase in pressure from 4000 to 5000 mbar) actually occur in all sorption curves. This microbalance is very sensitive. Its weighting resolution is ±0.2 μg. In turn, the temperature is stabilized with an accuracy of ±0.2 K and the pressure with an accuracy of ±0.02% of the measurement range. Therefore, the microbalance will record even a very small mass change associated with temperature and pressure fluctuations, once equilibrium has been reached at a given temperature and pressure.

**Figure 3:**
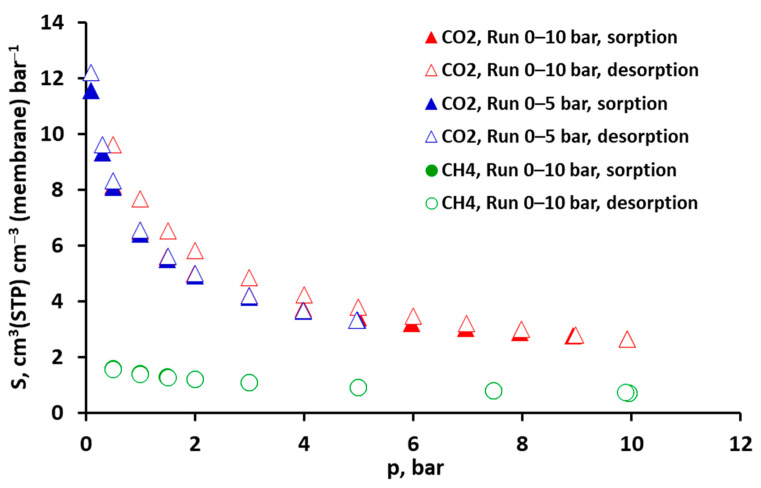
Experimental solubility of carbon dioxide and methane in the polysulfone-based membrane from Air Products’ PRISM PA1020–P1 module at 293 K.

**Figure 4:**
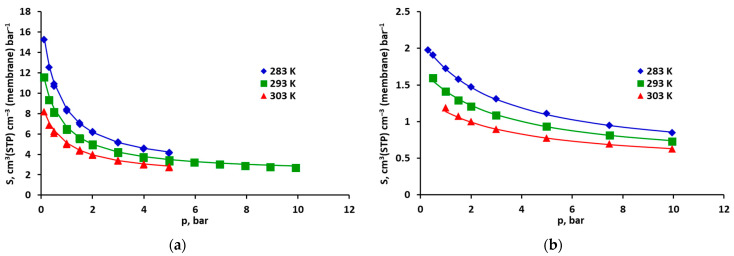
Solubility of pure (**a**) CO_2_ and (**b**) CH_4_ in the polysulfone-based membrane from Air Products’ PRISM PA1020–P1 module. Points represent experimental data and lines Dual Mode Sorption (DMS) model predictions (for minimized solubility squared differences).

The isotherms of CO_2_ and CH_4_ are shown in Figure 3 and Figure 4, expressed as solubilities. As can be seen in these figures, the solubility of pure CO_2_ is generally about two to six times higher than that of CH_4_. The greatest differences occur for lower pressures and, additionally, the isotherm of carbon dioxide is strongly nonlinear. In the case of methane (Figure 3) sorption and desorption, the points practically overlap in the entire pressure range. The same applies in principle to CO_2_, when the isotherm was measured in the pressure range of 0–5 bar. This suggests that the process is fully reversible in these cases, i.e., penetrants do not cause any observable and lasting changes in the membrane material. The similar solubility of carbon dioxide and methane in other polysulfone-based materials is presented in [23,24,25].

However, when the pressure range is extended to 10 bar in the case of CO_2_, a hysteresis is observed, i.e., the desorption points are located above the sorption points. This phenomenon indicates that equilibrium was not reached during desorption or during both sorption and desorption. As can be seen in Figure 2, equilibrium was established relatively quickly in all cases and the measurement time for a single isotherm point was sufficiently long. In this situation, it can be assumed that the appearance of hysteresis is related to the presence of another phenomenon that visibly accompanies the physical sorption/desorption.

In the case of the physical adsorption of gases or vapors in solids with a stable pore (voids) structure, hysteresis is a signal that condensation has occurred in the micropores. Typically, in such a situation, for the points on the lower hysteresis line (sorption points), equilibrium is reached, and the corresponding mass uptake curves look similar to those shown in Figure 3. However, for points on the upper hysteresis line (desorption), equilibrium is not reached within the given measurement time because the desorption kinetics are much slower than the sorption kinetics.

**Figure 5:**
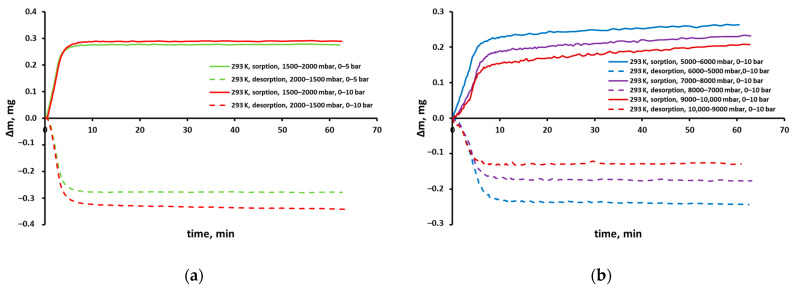
Mass change due to sorption (solid lines) and desorption (dashed lines) of CO_2_ at 293 K concerning (**a**) two isothermal runs in pressure range of 0–5 bar (green) and 0–10 bar (red), pressure below 5 bar, (**b**) isothermal run in pressure range of 0–10 bar, pressure above 5 bar.

A similar picture could be drawn for the CO_2_ isotherm in the polysulfone-based membrane determined in the pressure range of 0–10 bar, as is presented in Figure 5a. The figure shows the sample weight gain/loss due to the CO_2_ sorption/desorption between adjacent pressure points for several points of this isotherm. Therefore, when the pressure was changed from 1500 to 2000 mbar (solid red line) during the sorption stage, the sample weight increased by approximately 0.29 mg and then remained constant until the end of the measurement for that point. Virtually the same increase in the sample weight was recorded with such a pressure change when the CO_2_ isotherm was determined in the independent measurement in the pressure range of 0–5 bar (solid green line). In turn, when the pressure was changed from 2000 to 1500 mbar (dashed red line) during the desorption stage, the sample weight decreased relatively quickly by about 0.32 mg, and then it decreased systematically until the end of the measurement for this isotherm point. It was different in the case of the isotherm determined in the pressure range of 0–5 bar when the sample weight remained constant until the end of the measurement after an initial decrease of about 0.27 mg (dashed green line).

It should be emphasized here that in the case of the isotherm determined between 0 and 5 bar, the change in the sample weight between adjacent pressure points is similar in absolute value. This is shown, for example, in Figure 5a (green lines) and this means that equilibrium was also reached (i.e., the sorption/desorption was complete) for lower and higher pressures. However, the opposite is the case with the CO_2_ isotherm determined between 0 and 10 bar. As shown earlier, the sample mass loss during the desorption outweighs its increase during the sorption, when the pressure was varied between 1500 and 2000 mbar. This would mean that the desorption at higher pressures may have been difficult and incomplete.

Indeed, as can be seen in Figure 5b, at pressures higher than 5 bar, the mass loss during the desorption is lower than the mass gain during the sorption between corresponding adjacent pressure levels. The desorption is incomplete at these pressure levels, although the course of the mass uptake curves during the desorption (dashed lines in Figure 5b) would suggest that an equilibrium state has been reached. This is also evidenced by the fact that the desorption points are located higher than the sorption points on the isotherm graph (Figure 3). It should be noted, however, that in the case of the mass uptake curves recorded during the sorption (continuous lines in Figure 5b), the equilibrium is not achieved at pressures higher than 5 bar. After an initial, relatively rapid increase, the mass of CO_2_ in the polymer continues to increase monotonically until the end of the measurement for a given isotherm point. The latter observation suggests that there is a systematic increase in the sorption capacity of the tested polymer membrane sample, which means that it is most likely a swelling.

Both of these phenomena, the condensation of CO_2_ in glassy polymers and their swelling upon exposure to this gas when its pressure is high, are widely described in the literature [26,27]. Both are unfavorable from the point of view of the separation of gas mixtures containing carbon dioxide, such as biogas, using membrane methods. Condensation of carbon dioxide in the fractional free volume (FFV) of the glassy polymer may result in hindered desorption of CO_2_ from this region and, consequently, limited overall membrane permeability to this gas [28]. In turn, the swelling of the membrane worsens its selectivity, facilitating the transport of mixture components being separated from carbon dioxide (e.g., methane in the case of biogas) [12,28,29].

In the case considered in this paper, both of these phenomena already appear at a relatively low carbon dioxide pressure. However, this is not a problem from the point of view of the adsorptive–membrane process, which is related to the research presented in this paper. In this process, the partial pressure of CO_2_ does not exceed 5 bar [24,30,31]. Moreover, in real-world applications, membrane swelling should be avoided because the separation properties of the membrane would become unstable and unpredictable. Indeed, commercial membrane systems are dedicated to a specific process and specific process conditions. Only under such circumstances can the expected lifetime of the membrane system be guaranteed (e.g., 8–12 years). Therefore, in the further part of this work, the analysis was limited to the pressure range of 0–5 bar in the case of carbon dioxide, so as not to obscure the picture of the pure sorption–diffusion phenomena occurring in the tested membrane.

In the case of all the isotherms discussed above, including the one in which hysteresis occurred, the sample returned to its original state after regeneration, both in terms of its initial weight and the sorption capacity for CO_2_ and CH_4_. The sample regeneration method is presented in Section 3.2. Both identified phenomena, CO_2_ condensation in FFV (fractional free volume) and membrane swelling, are therefore reversible in the range of pressures and temperatures at which the research was carried out. This is consistent with the common view presented in the available literature regarding the influence of carbon dioxide on the condition of glassy membranes [28,32,33,34].

### 2.2. Solubility of Pure CO_2_, CH_4_ and Their Mixtures According to DMS Model

The sorption isotherms of carbon dioxide and methane in the polysulfone-based membrane sample were described using the Dual Mode Sorption (DMS) model. In this model, the simultaneous gas sorption in both fractions of membrane material is considered: Henry sorption in the matrix phase and Langmuir sorption in the microvoid region, called the fractional free volume (FFV). The model is presented in detail in Section 3.3. The fitting was performed using the nonlinear least-squares method, separately for the experimental points expressed as C = f(p) and S = f(p). In the first case, hereinafter referred to as the model with minimized concentration squared differences, each isotherm was approximated by Equation (4). In the second case, hereinafter referred to as the model with minimized solubility squared differences, the DMS model coefficients were estimated using Equation (5). In this way, two sets of k_D_, b and C’_H_ coefficients were obtained for each gas. Their temperature dependence is graphically presented in Figures S1–S3 (points) in the Supplementary Material. As can be observed from these figures, k_D_, b and C’_H_ decrease with increasing temperature, according to Equation (8), Equation (9) and Equation (10), respectively. The coefficients of these equations were also determined using the least-squares method and are summarized in Table 1.

It should be mentioned here that although the Dual Mode Sorption model is widely used and accepted for describing gas transport through polymer membranes, the issue of determining its coefficients is widely discussed in the literature [11,12,13,14,35]. One of the problems indicated is that different sets of model coefficients can be obtained for the same experimental data set with good numerical fitting accuracy [11,36,37,38,39]. It seems that an appropriate number of available experimental points determined in the appropriate range of the function argument (in this case pressure) does not guarantee obtaining DMS model parameter values that will well reflect the solubility in FFV and the polymer matrix. For gases such as CH_4_ or N_2_, solubility is low at low pressures. It changes nonlinearly with the pressure in this region, and at the same time, the number of experimental points that can be determined with sufficient accuracy is limited. This uncertainty in the determination of solubility is independent of whether it is determined indirectly, based on experimentally determined permeability and diffusivity, or directly, as in the present work. It is also reflected in the values of the b and C’_H_ coefficients, which describe the sorption in the Langmuir area. In the case of carbon dioxide sorption at higher pressures, swelling of the membrane, as discussed above, or plasticization [32,34,37,38] can occur. These phenomena directly affect the k_D_ value, which reports the amount of penetrant absorbed in the polymer matrix. In the present study, the physical significance of the obtained coefficients of the DMS model was evaluated based on several independent premises.

The sorption isotherms of CO_2_ and CH_4_ determined using the DMS model with the coefficients from Table 1 are presented graphically (as lines), together with the experimental points, in Figure 4a and Figure 4b, respectively, for the case with minimized solubility squared differences and in Figure S4 in the Supplementary Material for the case with minimized concentration squared differences.

As can be seen in these figures, the qualitative agreement between the experimental isotherms and those determined by the DMS model is good for both gases and both methods of approximating the model coefficients. The quantitative agreement is also good, as indicated by the low values of the mean relative error (RE) and standard error of the estimate (SEE) given in Table 1. These errors are defined by Equations (15) and (16), respectively. The latter was proposed to assess the quality of the DMS model fit in [11]. As can be seen in Figures S1–S3 in the Supplementary Material, both approximation methods result in similar sets of k_D_, b and C’_H_ coefficients for both gases. Table 1 also lists the values of the C’_H1_/−C’_H0_ ratio, i.e., the theoretical intersection of the C’_H_ = f(T) line with the abscissa axis. This value refers to the temperature at which the fractional free volume in the polymer should theoretically disappear [14]. The temperature is similar in each case, which can be considered as additional evidence of the good quality of the model fit to the experimental results, as well as the consistency of the obtained results. Thus, it can be stated that the conclusions and observations presented in the following sections regarding the solubility of the CH_4_/CO_2_ mixture components in the tested polysulfone-based membrane sample are independent of how the model coefficients are approximated.

**Figure 6:**
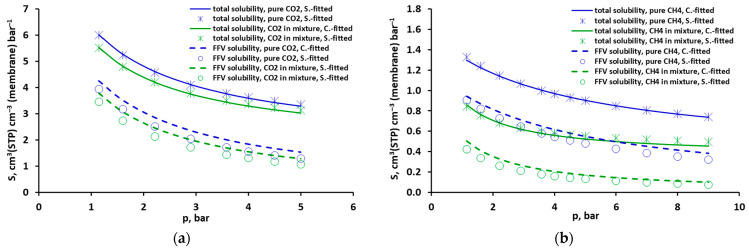
Total and FFV solubility of (**a**) CO_2_ and (**b**) CH_4_, pure and mixed (CO_2_: 50 vol.%/CH_4_: 50 vol.%) at 295 K according to Dual Mode Sorption (DMS) model predictions for minimized concentration (blue) and solubility (green) squared differences.

The solubility of carbon dioxide and methane, pure and mixed (CO_2_: 50 vol.%/CH_4_: 50 vol.%), as a function of gas partial pressure, determined from the DMS model for a temperature of 295 K is presented in Figure 6. The same was performed for the mixture of CO_2_: 40 vol.%/CH_4_: 60 vol.% (cf. Figure S5 in the Supplementary Material). The mixture’s composition, temperature and pressure ranges correspond to the experimental conditions in which the permeation coefficients of both gases, pure and mixed, were determined [3,21]. As can be seen in Figure 6 and Figure S5 in the Supplementary Material, the value of total solubility does not depend on how the coefficients of the DMS model are approximated, both for pure and mixed gases. However, the model with minimized concentration squared differences, on average, gives several percent higher FFV solubility values than the one with minimized solubility squared differences.

**Figure 7:**
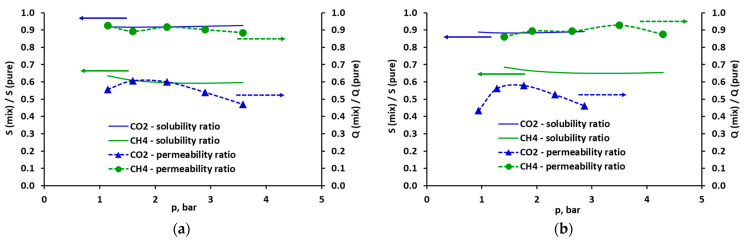
Comparison of solubility (solid lines) and permeance (solid points with dotted lines) of pure and mixed CO_2_ (blue) and CH_4_ (green) for the mixture of (**a**) CO_2_ (50 vol.%)/CH_4_ (50 vol.%) and (**b**) CO_2_ (40 vol.%)/CH_4_ (60 vol.%). Solubility was calculated using the DMS model with minimized solubility squared differences.

The total solubility of pure methane is about 4–5 times lower than that of pure carbon dioxide. For both gases, the fraction of solubility in the Langmuir area is significant and decreases with increases in pressure. In the pressure range of 1–5 bar, it decreases from 65% to 46% and from 73% to 43%, respectively, for CO_2_ and CH_4_ (in the case of minimized solubility squared differences). In the case of mixed gases, the presence of methane has only a slight effect on CO_2_ solubility in the FFV of the studied polysulfone-based membrane, and consequently on its total solubility (Figure 6a). As can be seen in Figure 7, the ratio of pure and mixed carbon dioxide solubility is in the range of 0.89–0.93. On the other hand, the total solubility of methane significantly decreases in the presence of carbon dioxide due to a significant reduction in its solubility in the fractional free volume. In this case, the ratio of pure and mixed CH_4_ solubility is in the range of 0.59–0.69 (Figure 7). This clearly better affinity of carbon dioxide than methane during FFV adsorption is expressed by higher values of the parameter b, which are in the range of 1.22–1.61 bar^−1^ and 0.31–0.33 bar^−1^ for CO_2_ and CH_4_, respectively (the model with minimized solubility squared differences, cf. Figure S3 in the Supplementary Material). The heat of adsorption in FFV is also significantly higher for CO_2_, as expressed by the −ΔH_L_/R parameter. In the case of minimized solubility squared differences, it is equal to 1194.6 K^−1^ for CO_2_ and 305.5 K^−1^ for CH_4_ (cf. Table 1).

The comparison of the solubility of pure and mixed gas, as well as the permeability of pure and mixed CO_2_ and CH_4_, is presented in Figure 7. The solubility was determined using the DMS model with minimized solubility squared differences. The similar plots obtained using the DMS model with minimized concentration squared differences are presented in Figure S6 in the Supplementary Material. The permeability coefficients determined experimentally in [3,21] are listed in Tables S1 and S3 in the Supplementary Material, and their corresponding ratios are marked in Figure 7 and Figure S6 in the Supplementary Material by points connected by dotted lines. As can be seen in these figures, the better affinity of CO_2_ towards the tested polysulfone membrane does not clearly imply the preference of this gas during the separation of the CO_2_/CH_4_ mixture. For both gases, the permeability coefficients of mixed gas are lower than in the case of pure gas. However, while the permeability of methane in the mixture is only slightly less than that of pure gas, the permeability of carbon dioxide is much less in the CO_2_/CH_4_ mixture than that of pure gas. The permeability ratio of mixed and pure CO_2_ ranges from 0.43 to 0.61, while for methane it is 0.86 to 0.93 (Figure 7). Moreover, the permeability coefficient of carbon dioxide in the CO_2_/CH_4_ mixture varies significantly with pressure, reaching a maximum value at a pressure of about 1.8 bar. The phenomenon of a decrease in CO_2_ flux and unchanging CH_4_ flux is often reported in the literature for the separation of CO_2_/CH_4_ mixtures in glassy polymer membranes. This phenomenon cannot be explained by the competitive sorption in FFV. Carbon dioxide is the dominant component in terms of its solubility in both the FFV and the polymer matrix, causing a significant reduction in the solubility of methane. This is also frequently reported [28,36,39,40], therefore the mentioned phenomenon is often explained by the fact that CO_2_ present in the polymer matrix facilitates the diffusion of methane, pushing the polymer chains apart [28,36,40].

### 2.3. Selectivity and Diffusivity of Pure CO_2_, CH_4_ and Their Mixtures

**Figure 8:**
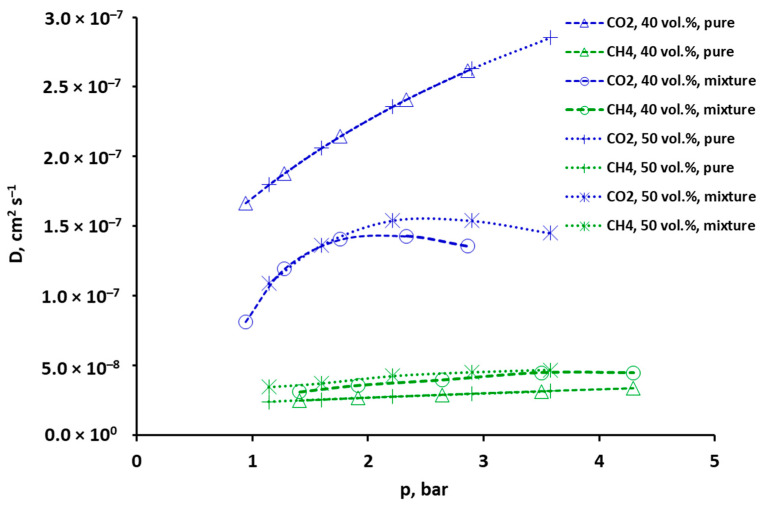
Comparison of pure and mixed diffusivity at 295 K for CO_2_ (blue color) and CH_4_ (green color) in the case of the DMS model with minimized solubility squared differences.

The simple relation between the permeability (or permeance), solubility and diffusivity provided by the solution–diffusion model (Equation (1)) allows one of these quantities to be determined, once the two others (usually including the permeability/permeance) have been directly measured [32,38,39,40,41]. In the present study, the diffusion coefficients of methane and carbon dioxide, pure and mixed, were determined at 295 K based on the solubility and permeance of these gases in the polysulfone-based membrane sample. Their dependence on partial pressure is presented graphically in Figure 8 (as well as in Figure S7 in the Supplementary Material) and the values are given in Tables S2 and S4 in the Supplementary Material. The points in these figures correspond to the experimentally determined permeances, and the dotted lines are drawn to guide the eye. As can be noticed in Figure 8 and Figure S7 in the Supplementary Material, the diffusion coefficients of pure CO_2_ and pure and mixed methane increase monotonically and almost linearly with an increase in partial pressure. The diffusion of CH_4_ in the mixture with CO_2_ is slightly faster than that of the pure gas, with the points of both CO_2_ concentrations (40 and 50 vol.%) practically aligning. In contrast, the diffusion of carbon dioxide is noticeably slower in the presence of methane. The diffusion coefficient of this gas initially increases with pressure to begin a slight decrease after reaching a maximum value. And near this maximum, the lines corresponding to both mixtures diverge.

**Figure 9:**
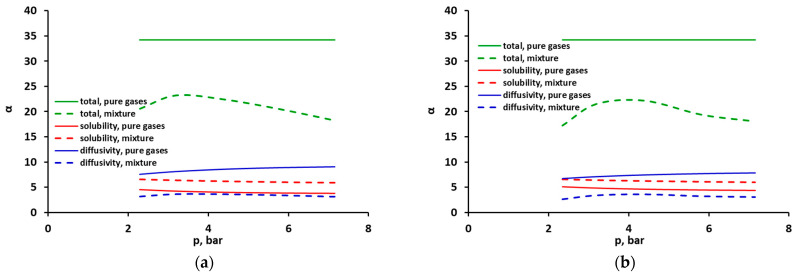
Comparison of total (green), solubility (red) and diffusivity (blue) CO_2_ vs. CH_4_ selectivity, pure (solid lines) and mixed (dashed lines), for the mixture of (**a**) CO_2_ (50 vol.%)/CH_4_ (50 vol.%) and (**b**) CO_2_ (40 vol.%)/CH_4_ (60 vol.%). The case of the DMS model with minimized solubility squared differences.

The interaction between carbon dioxide and methane in the glassy polymer is, of course, reflected in the selectivity (defined by Equation (2)), which is an important indicator that determines the efficiency of the separation process. As can be seen in Figure 9 (and also in Figure S8 in the Supplementary Material), the overall permselectivity of CO_2_ vs. CH_4_ in the mixture in the tested polysulfone membrane is clearly lower than in the case of pure components (the ideal selectivity). It is true that there is an improvement in solubility selectivity in the mixture due to the dominance of CO_2_ during adsorption in FFV. However, because of the decrease in the diffusion coefficient of this gas in the mixture, the diffusivity selectivity also decreases dramatically, which is a critical factor in the overall deterioration of the separation conditions for CO_2_/CH_4_ mixtures. The numerical values of the selectivity coefficients are presented in Tables S1–S4 in the Supplementary Material.

The experimentally determined diffusion coefficients of CO_2_ and CH_4_ are described using the partial immobilization model, presented in Section 3.3. The linearized form of Equations (11) and (12), for pure and mixed gases, respectively, are shown graphically in Figures S9–S12 in the Supplementary Material. In this approach, the D_D_ coefficient, i.e., the diffusivity in the polymer matrix, is the slope of the straight line, and the ratio F of the diffusion coefficients in the Langmuir and Henry regions can be determined from the intercept (D_D_∙K∙F). Both parameters are presented in Table 2 for CO_2_ and CH_4_, pure and mixed, with both ways of approximating the DMS model coefficients. The average relative error in determining the diffusion coefficients of carbon dioxide and methane in the mixture was 11.8% and 5.6%, respectively, for fitting the DMS model coefficients with minimized solubility squared differences, and 19.2% and 14, 2% for the case with minimized concentration squared differences. Thus, in the former case, the accuracy of fitting the overall diffusion coefficient is clearly better, but the values of D_D_ and F are similar for both ways of approximating the DMS model coefficients.

As can be seen in Table 2, for CH_4_, in a mixture with carbon dioxide, the mobility in the FFV disappears (F = 0). On the other hand, the presence of methane induces the mobility of carbon dioxide in the fractional free volume (F increases from about 0 for pure CO_2_ to about 0.29 or 0.35 for the mixture).

A comparison of the permeability of methane and carbon dioxide and their solubility in FFV and in the polymer matrix leads to the obvious conclusion that the former has a much lower affinity towards the tested glassy polymer. Thus, on the surface of the polymer voids, methane is likely to form a monolayer and fill the rest of the FFV while remaining in the gas phase. This would explain its mobility when it permeates alone. As a result, from the previous considerations, in the presence of CO_2_, methane can still occupy parts of the FFV surface, but it is probably no longer present there in the non-adsorbed phase, being displaced by carbon dioxide. In the case of the latter, the lack of mobility in the FFV in the pure state, with the strong affinity shown above towards the tested membrane, additionally indicates the possibility of CO_2_ condensation. On the other hand, the revealing of CO_2_ mobility in the FFV in the presence of methane may suggest that some carbon dioxide remains in the gas phase in this area.

When CH_4_ permeates together with carbon dioxide, its diffusivity in the polymer matrix is increased (by about 6% in the case with minimized solubility squared differences). This observation would confirm the hypothesis, based on other premises, that CO_2_ facilitates methane transport in the membrane matrix because it pushes the polymer chains apart [11,39,40]. On the other hand, for carbon dioxide, the presence of methane means about a two-fold decrease in its diffusivity in the polymer matrix. It can be assumed that the presence of slower diffusing gas (methane) slows down the diffusion of faster diffusing gas (carbon dioxide), as was observed in [42].

It should be noted here that the partial immobilization model does not predict phenomena that may accompany CO_2_ permeation through polymer membranes, such as swelling or plasticization [12,13,28,38]. In particular, the D_D_ coefficient is a constant parameter in this model, and the dependence of the overall diffusion coefficient (D) on the concentration (in this case, the partial pressure) is captured by the product (Equation (11)) or sum of the products (Equation (12)) of the parameter b and the partial pressure. For gases that adsorb strongly (in this case, CH_4_) or very strongly (in this case, CO_2_) in the FFV, the overall value of the diffusion coefficient will theoretically tend toward D_D_. Thus, in the case of changes in the diffusivity of CH_4_/CO_2_ mixture components compared to pure gases, it is only possible to indicate the observed trends without explicitly explaining them based on the theoretical description that is often used and also applied in this work.

As can be concluded from Equation (12), in the presence of strongly adsorbing CO_2_, the total CH_4_ diffusion coefficient is close to the value D_D_ and greater than in the case of pure gas. And this increase actually compensates for the decrease in methane solubility and keeps the CH_4_ permeance in the mixture at a practically constant level compared to pure gas. It seems that when the diffusion of pure methane in the polymer matrix is hindered, it will tend to remain in the FFV (the coefficient F in Equation (11) is greater than zero for pure CH_4_, cf. Table 2). Carbon dioxide, by pushing methane out of the FFV, probably additionally forces its diffusion through the polymer matrix up to the D_D_ limit.

## 3. Materials, Methods and Model

### 3.1. Membrane Sample and Gases

The sample of a modified polysulfone membrane for sorption experiments was derived from the hollow-fiber PRISM PA1020 module, provided by Air Products Inc., Westminster, MD, USA. The fibers were cut into pieces about 2 mm in length, forming a sample with an initial weight of 154.2 mg. The module is used mainly for the separation of air. Its maximal working temperature and pressure is 328 K and 15 bar, respectively.

Methane of at least 99.995% purity was supplied by Messer Polska Sp. z o.o., Chorzów, Poland, while carbon dioxide of at least 99.95% purity was supplied by Siad Poland Sp. z o.o., Ruda Śląska, Poland and helium of at least 99.9999% purity was supplied by Air Liquide Polska Sp. z o.o., Dąbrowa Górnicza, Poland.

### 3.2. Sorption Experiments

The sorption isotherms of carbon dioxide and methane were determined based on gravimetric measurements using a microbalance (IGA003, Hiden Isochema Ltd., Warrington, UK) with a resolution of 0.2 μg and the buoyancy force correction. The microbalance is fully thermostatted to eliminate the effect of ambient temperature (the temperature variations in the system do not exceed ±0.2 K). The measurements were conducted in the static mode, i.e., the sample container was filled with gas until a desired pressure was reached, and then the gas supply was cut off until the equilibrium was established. The IGA microbalance is described in more detail in reference [43].

Before starting the measurements, the sample was degassed under a vacuum for 24 h, and then its density was measured. This measurement was performed in helium, with a step change in the pressure of 500 mbar over a range of 2–18 bar and the temperature kept constant at 273 K. Thus, the determined density was equal to 1.229 g cm^−3^. The sample was fully degassed before each temperature and gas change.

The isotherms of the CO_2_ and CH_4_ sorption at a given temperature (283, 293 or 303 K) were determined in two runs: the sorption and desorption, when the pressure was increased and decreased, respectively, with a given step. The pressure range was 0–10 bar for methane and 0–5 bar for carbon dioxide. The additional isotherm of CO_2_ was also measured at 293 K in the pressure range of 0–10 bar. The measurement of a given isotherm point ended when the measured change in the sample mass reached 99.8% of the predicted asymptotic value or the measurement time for a given point exceeded 120 min.

### 3.3. Dual Mode Sorption Model

In order to theoretically describe the sorption isotherms, the Dual Mode Sorption model was assumed [11,12,13,14,35], in which the concentration of a gas i-th in a glassy polymer embraces two fractions. The first one (C_Di_) concerns gas dissolved in the polymer matrix and is described by Henry’s law. The other (C_Hi_) relates to the gas fraction adsorbed in the fractional free volume (FFV) of the glassy polymer and is described by the Langmuir model. The sum of both contributions can be defined as follows:

(4) $$C_{i}=C_{Di}+C_{Hi}=k_{Di}p_{i}+C_{Hi}^{\prime }\frac{b_{i}p_{i}}{1+b_{i}p_{i}}$$

where k_D_ is Henry’s solubility coefficient (cm^3^(STP) cm^−3^ bar^−1^), b is the Langmuir affinity coefficient (bar^−1^) and C’_H_ is the Langmuir saturation constant (cm^3^(STP) cm^−3^). In the above formula and when presenting experimental data, the partial pressure is used instead of fugacity like in several other works [20,44], since in the pressure and temperature range used in this work the assumption of ideal gas behavior is valid [37]. Although, due to the different departures from ideality of CO_2_ and CH_4_, fugacities (i.e., the effective pressure of a non-ideal gas) are often used to describe the data in mixed-gas sorption measurements [11]. It is true, that the values of the DMS model parameters obtained using pressure or fugacity may be different; however, often the calculations have the same accuracy and lead to similar results, as reported in the literature [45,46]. Moreover, based on calculations presented in [37], one can notice that in the pressure range of 0–5 bar, which was used in our studies, the non-ideality of CO_2_ and CH_4_ is of minor importance and, moreover, the gases show equal departures from ideality.

A simple transformation of Equation (4) with Equation (3) leads to the following expression describing the solubility S (cm^3^(STP) cm^−3^ bar^−1^):

(5) $$S_{i}=k_{Di}+C_{Hi}^{\prime }\frac{b_{i}}{1+b_{i}p_{i}}$$

In the case of gaseous mixtures Equations (4) and (5) take the following form [11,17,37]:

(6) $$C_{i}=k_{Di}p_{i}+C_{Hi}^{\prime }\frac{b_{i}p_{i}}{1+\sum_{j}b_{j}p_{j}}$$

(7) $$S_{i}=k_{Di}+C_{Hi}^{\prime }\frac{b_{i}}{1+\sum_{j}b_{j}p_{j}}$$

It is assumed that the parameters in the above formulas are independent of the presence of other mixture components. Thus, they are the same for both single- and mixed-gas sorption in glassy polymers. Consequently, penetrants do not affect each other during multicomponent sorption in the polymer matrix where Henry’s law is valid. The opposite is the case with FFV, where the presence of other mixture components will generally result in reduced adsorption of a given penetrant. Moreover, in this competition for adsorption sites in FFV, components with a higher Langmuir affinity coefficient b will prevail.

The parameters of DMS model equations are temperature-dependent. The temperature dependence of k_Di_ and b_i_ may be given as an Arrhenius-type relationship [11,12,47]:

(8) $$k_{Di}=k_{D0i}exp\left(\frac{-∆H_{Di}}{RT}\right)$$

(9) $$b_{i}=b_{0i}exp\left(\frac{-∆H_{Li}}{RT}\right)$$

where k_D0i_ and b_0i_ are pre-exponential coefficients in cm^3^(STP) cm^−3^ bar^−1^ and bar^−1^, respectively, ΔH_Di_ is the heat of sorption in Henry’s region, ΔH_Li_ is the heat of adsorption in FFV in kJ mol^−1^, T is the temperature in K and R is gas constant in kJ mol^−1^ K^−1^. The temperature dependence of C’_H_ is empirical; however, this parameter is related to the specific volume of FFV in the polymer. Since the latter changes linearly with temperature [14], a linear relationship between C’_H_ and T is assumed:

(10) $$C_{Hi}^{\prime }=C_{H0i}^{\prime }T+C_{H1i}^{\prime }$$

where parameters C’_H0i_ and C’_H1i_ are in cm^3^(STP) cm^−3^ bar^−1^ and cm^3^(STP) cm^−3^, respectively. It is expected that the C’_H_ value decreases with increasing experimental temperature [14] until it reaches 0 at T = -C’_H1i_/C’_H0i_, which may be interpreted as a transition of the polymer from a glassy to a rubbery state.

Assuming the validity of Fick’s law in the solution–diffusion and DMS models, the diffusion coefficient may be derived in the following form [14], successively for pure gases (Equation (11)) and mixtures (Equation (12)):

(11) $$D_{i}=D_{Di}\left[\frac{1+\frac{F_{i}K_{i}}{{\left(1+b_{i}p_{i}\right)}^{2}}}{1+\frac{K_{i}}{{\left(1+b_{i}p_{i}\right)}^{2}}}\right]=D_{Di}\left[\frac{{\left(1+b_{i}p_{i}\right)}^{2}+F_{i}K_{i}}{{\left(1+b_{i}p_{i}\right)}^{2}+K_{i}}\right]$$

(12) $$D_{i}=D_{Di}\left[\frac{{\left(1+\sum_{j=1}^{2}b_{j}p_{j}\right)}^{2}+F_{i}K_{i}}{{\left(1+\sum_{j=1}^{2}b_{j}p_{j}\right)}^{2}+K_{i}}\right]$$

where K is a dimensionless parameter, depending on temperature, which describes a relation between sorption in the Henry and Langmuir regions:

(13) $$K_{i}=\frac{C_{Hi}^{\prime }b_{i}}{k_{Di}}$$

In turn, dimensionless constant F is the ratio of the diffusion coefficient due to the Henry mode species (D_D_) and the diffusion coefficient due to the Langmuir mode species (D_H_).

(14) $$F_{i}=\frac{D_{Hi}}{D_{Di}}$$

The set of Equations (11)–(14) is referred to in the literature [14] and also in this work as the partial immobilization model.

The parameters of the DMS model for the sorption of CO_2_ and CH_4_ in the sample of a polysulphone-based membrane were determined independently at each temperature based on the experimental isotherms and using nonlinear regression. Since these parameters are sensitive to the parameter estimation methodology [11,36,37,38,39], the concentration and the solubility squared differences were minimized, using Equation (4) or Equation (5), respectively. Then, every set of k_Di_, b_i_ and C’_Hi_ was regressed over the experimental temperature range, using Equation (8), Equation (9) and Equation (10), respectively. The quality of the fit was assessed using average relative error (RE) and standard error of the estimate (SEE) [11], defined successively as follows:

(15) $$RE=\frac{\sum_{j}\frac{\left|y_{j,exp}-y_{j,calc}\right|}{y_{j,exp}}}{n}$$

(16) $$SEE=\sqrt{\frac{\sum_{j}{\left(y_{j,exp}-y_{j,calc}\right)}^{2}}{n-p}}$$

where y_j,exp_ is an experimental point j, y_j,calc_ is the value of the corresponding point calculated with the model, n is the number of experimental points used in the regression, and p is the number of model parameters.

## 4. Conclusions

This paper analyzes the factors influencing the mass transport of carbon dioxide and methane, in pure and mixed states, in a glassy polysulfone-based membrane from the Air Products’ PRISM PA1020–P1 module, used in a developed hybrid adsorptive–membrane process for biogas separation. It was found that the solubility of pure CO_2_ and CH_4_ in a sample of this membrane, determined gravimetrically in the temperature range of 283–303 K, is similar to that of other polysulfone membranes, and the effect of membrane swelling caused by the sorption of carbon dioxide is already observed for pressures higher than 5 bar. The experimental sorption isotherms of both gases were used to determine the coefficients of the Dual Mode Sorption model, which enables theoretical prediction of the solubility of CO_2_ and CH_4_ in the pure and mixed state. The physical plausibility of the model coefficients was justified on the basis of such premises as their consistency for different methods of fitting experimental data and models and good accuracy of the fit. Moreover, the analysis of the solubility of CO_2_ and CH_4_ indicated, among other things, the dominant share of sorption of both gases in the fractional free volume (FFV) of the tested polysulfone-based membrane and the much better solubility of CO_2_ in this membrane in the polymer matrix and in FFV, both pure and in a mixture with CH_4_.

On the basis of the solubility of CO_2_ and CH_4_, calculated at a temperature of 295 K from the DMS model, and the permeance of these gases measured in independent tests, their diffusion coefficients were determined in the sample of the tested polysulfone-based membrane, both in the pure and mixed state. These diffusivities were described by the partial immobilization model. It was found that the diffusion of CO_2_ (pure and mixed) in the tested sample of the glassy polymer is an order of magnitude faster than in the case of methane. It was also found that the presence of carbon dioxide promotes the transport of methane in the membrane, slightly accelerating its diffusion. At the same time, however, methane causes the CO_2_ diffusion rate to decrease by about two-fold. It was also observed that in the case of a mixture and in relation to pure gases, in the fractional free volume, the mobility of methane disappears and the mobility of CO_2_ appears.

A methodology has been developed to predict the values of the real transport coefficients of the mixture components, which is based on the DMS model and the experimentally determined solubility and diffusion of CH_4_ and CO_2_ in the glassy membrane material. The coefficients of the DMS and partial immobilization models determined in this work, taking into account the known limitations of these models, will be used to determine the permeance of CO_2_/CH_4_ mixture components. These data are essential for the simulation, optimization and scaling-up of the developed biogas separation process into bio-CH_4_ and bio-CO_2_.

## Figures and Tables

**Table 1:** DMS model coefficients in the case of sorption of CO_2_ and CH_4_ in the polysulfone-based membrane from the Air Products’ PRISM PA1020–P1 module.

	DMS Model with Minimized Concentration Squared Differences		DMS Model with Minimized Solubility Squared Differences	
Gas	CO_2_	CH_4_	CO_2_	CH_4_
k_D0_^1^	2.994 × 10^−3^	2.622 × 10^−2^	1.567 × 10^−2^	3.012 × 10^−2^
−ΔH_D_/R ^2^	1878.9	766.4	1439.6	776.9
C’_H0_^3^	−0.136	−0.096	−0.175	−0.093
C’_H1_^4^	49.33	33.41	58.95	31.39
b_0_^5^	9.997 × 10^−4^	1.427 × 10^−2^	2.363 × 10^−2^	1.122 × 10^−1^
−ΔH_L_/R ^2^	2025.1	825.8	1194.6	305.5
C’_H1_/−C’_H0_^6^	363.8	348.2	337.4	337.0
RE ^7^	1.51	1.41	2.1	0.97
SEE ^8^	0.182	0.039	0.173	0.021

^1^ k_D0_ is in cm^3^(STP) cm^−3^ (membrane) bar^−1^; ^2^ −ΔH_D_/R and −ΔH_L_/R is in K; ^3^ C’_H0_ is in cm^3^(STP) cm^−3^ (membrane) K^−1^; ^4^ C’_H1_ is in cm^3^ (STP) cm^−3^ (membrane); ^5^ b_0_ is in bar^−1^; ^6^ C’_H1_/−C’_H0_ is in K; ^7^ RE (average relative error) is in %; ^8^ SEE is in cm^3^(STP) cm^−3^ (membrane) and in cm^3^(STP) cm^−3^ (membrane) bar^−1^ for the concentration and solubility squared differences, respectively.

**Table 2:** Coefficients of the partial immobilization model in the case of sorption and diffusion of CO_2_ and CH_4_ in the polysulfone-based membrane from Air Products’ PRISM PA1020–P1 module. 295 K.

	DMS Model with Minimized Concentration Squared Differences				DMS Model with Minimized Solubility Squared Differences			
	Pure		Mixture		Pure		Mixture	
Gas	CO_2_	CH_4_	CO_2_	CH_4_	CO_2_	CH_4_	CO_2_	CH_4_
K	5.113	3.398	5.113	3.398	4.873	2.947	4.873	2.947
D_D_^1^	3.53 × 10^−7^	5.11 × 10^−8^	1.7 × 10^−7^	5.29 × 10^−8^	3.21 × 10^−7^	4.58 × 10^−8^	1.6 × 10^−7^	4.86 × 10^−8^
F	0.069	0.226	0.345	0	0	0.211	0.291	0

^1^ D_D_ is in cm^2^ s^−1^.

## Data Availability

Data are contained within the article and Supplementary Materials.

## Supplementary Materials

The following supporting information can be downloaded at: www.mdpi.com/article/10.3390/molecules30030614/s1, Table S1: Summary of the permeances of CO_2_ and CH_4_, pure and in the mixture of 50% CO_2_/50% CH_4_, at 295 K in the polysulfone-based membrane from Air Products’ PRISM PA1020–P1 module; Table S2: Summary of the solubility, diffusivity and selectivity of CO_2_ and CH_4_, pure and in the mixture of 50% CO_2_/50% CH_4_, at 295 K in the polysulfone-based membrane from Air Products’ PRISM PA1020-P1 module; Table S3: Summary of the permeances of CO_2_ and CH_4_, pure and in the mixture of 40% CO_2_/60% CH_4_, at 295 K in the polysulfone-based membrane from Air Products’ PRISM PA1020-P1 module; Table S4: Summary of the solubility, diffusivity and selectivity of CO_2_ and CH_4_, pure and in the mixture of 40% CO_2_/60% CH_4_, at 295 K in the polysulfone-based membrane from Air Products’ PRISM PA1020–P1 module; Figure S1: Temperature dependence of Henry’s constant in the Dual Mode Sorption (DMS) model for (a) CO_2_ and (b) CH_4_. The dotted lines are from the fit; Figure S2: Temperature dependence of the Langmuir adsorption capacity in the Dual Mode Sorption (DMS) model for (a) CO_2_ and (b) CH_4_. The dotted lines are from the fit; Figure S3: Temperature dependence of the Langmuir affinity constant in the Dual Mode Sorption (DMS) model for (a) CO_2_ and (b) CH_4_. The dotted lines are from the fit; Figure S4: Temperature dependence of the Langmuir affinity constant in the Dual Mode Sorption (DMS) model for (a) CO_2_ and (b) CH_4_; Figure S5: Concentration of pure (a) CO_2_ and (b) CH_4_ in the polysulfone-based membrane from Air Products’ PRISM PA1020–P1 module. Points represent experimental data and lines Dual Mode Sorption (DMS) model predictions (for minimized concentration squared differences); Figure S6: Comparison of solubility (solid lines) and permeance (solid points with dotted lines) of pure and mixed CO_2_ (blue) and CH_4_ (green) for the mixture of (a) CO_2_ (50 vol.%)/CH_4_ (50 vol.%) and (b) CO_2_ (40 vol.%)/CH_4_ (60 vol.%). Solubility calculated using the DMS model with minimized concentration squared differences; Figure S7: Comparison of pure and mixed diffusivity at 295 K for CO_2_ (blue color) and CH_4_ (green color) in the case of the DMS model with minimized concentration squared differences; Figure S8: Comparison of total (green), solubility (red) and diffusivity (blue) CO_2_ vs. CH_4_ selectivity, pure (solid lines) and mixed (dashed lines), for the mixture of (a) CO_2_ (50 vol.%)/CH_4_ (50 vol.%) and (b) CO_2_ (40 vol.%)/CH_4_ (60 vol.%). The case of the DMS model with minimized concentration squared differences; Figure S9: Diffusivity of (a) pure and (b) mixed CO_2_ in the polysulfone-based membrane from Air Products’ PRISM PA1020–P1 module at 295 K according to the linearized partial immobilization model. A straight dotted line is from the fit. The case of the DMS model with minimized solubility squared differences; Figure S10: Diffusivity of (a) pure and (b) mixed CH_4_ in the polysulfone-based membrane from Air Products’ PRISM PA1020-P1 module at 295 K according to the linearized partial immobilization model. A straight dotted line is from the fit. The case of the DMS model with minimized solubility squared differences; Figure S11: Diffusivity of (a) pure and (b) mixed CO_2_ in the polysulfone-based membrane from Air Products’ PRISM PA1020–P1 module at 295 K according to the linearized partial immobilization model. A straight dotted line is from the fit. The case of the DMS model with minimized concentration squared differences; Figure S12: Diffusivity of (a) pure and (b) mixed CH_4_ in the polysulfone-based membrane from Air Products’ PRISM PA1020–P1 module at 295 K according to the linearized partial immobilization model. A straight dotted line is from the fit. The case of the DMS model with minimized concentration squared differences.
