# Supplementary material for: Solubility and Diffusion of Main Biogas Components in a Glassy Polysulfone-Based Membrane
Source: Molecules. 2025 Jan 30;30(3):614. doi: 10.3390/molecules30030614 (PMC11821198; doi:10.3390/molecules30030614)
Supplement: Supplementary file 1 [file molecules-30-00614-s001.zip › molecules-3415287-supplementary.pdf]

Supplementary Material (ESI)

Solubility and diffusion of main biogas components in a glassy polysulfone-based membrane

Marek Tańczyk [1,] *, Aleksandra Janusz-Cygan [1], Anna Pawlaczyk-Kurek [1], Łukasz Hamryszak [1], Jolanta Jaschik [1] and Katarzyna Janusz-Szymańska [2]

1 Institute of Chemical Engineering, Polish Academy of Sciences, Bałtycka 5, 44-100 Gliwice, Poland; ajcygan@iich.gliwice.pl; ania.pawlaczyk@iich.gliwice.pl; lukasz.hamryszak@iich.gliwice.pl; jjaschik@iich.gliwice.pl

2 Department of Power Engineering and Turbomachinery, Faculty of Energy and Environmental Engineering, Silesian University of Technology, Konarskiego 18, 44-100 Gliwice, Poland; katarzyna.janusz-szymanska@polsl.pl

* Correspondence: mtanczyk@iich.gliwice.pl; Tel.: +48 32 234 69 15

THE TABLE OF CONTENTS

Table S1. Summary of the permeances of CO_2 and CH_4, pure and in the mixture of 50% CO_2 / 50% CH_4, at 295 K in the polysulfone-based membrane from Air Products' PRISM PA1020-P1 module. 3

Table S2. Summary of the solubility, diffusivity and selectivity of CO_2 and CH_4, pure and in the mixture of 50% CO_2 / 50% CH_4, at 295 K in the polysulfone-based membrane from Air Products' PRISM PA1020-P1 module............ 3

Table S3. Summary of the permeances of CO_2 and CH_4, pure and in the mixture of 40% CO_2 / 60% CH_4, at 295 K in the polysulfone-based membrane from Air Products' PRISM PA1020-P1 module. 4

Table S4. Summary of the solubility, diffusivity and selectivity of CO_2 and CH_4, pure and in the mixture of 40% CO_2 / 60% CH_4, at 295 K in the polysulfone-based membrane from Air Products' PRISM PA1020-P1 module............ 4

Fig. S1. Temperature dependence of the Henry's constant in the Dual Mode Sorption (DMS) model for (a) CO_2 and (b) CH_4. The dotted lines are from the fit. 5

Fig. S2. Temperature dependence of the Langmuir adsorption capacity in the Dual Mode Sorption (DMS) model for (a) CO_2 and (b) CH_4. The dotted lines are from the fit............ 5

Fig. S3. Temperature dependence of the Langmuir affinity constant in the Dual Mode Sorption (DMS) model for (a) CO_2 and (b) CH_4 The dotted lines are from the fit. 5

Fig. S4. Temperature dependence of the Langmuir affinity constant in the Dual Mode Sorption (DMS) model for (a) CO_2 and (b) CH_4............ 6

Fig. S5. Concentration of pure (**a**) CO_2 and (**b**) CH_4 in the polysulfone-based membrane from Air Products' PRISM PA1020-P1 module. Points represent experimental data and lines Dual Mode Sorption (DMS) model predictions (for minimized the concentration squared differences). 6

Fig. S6. Comparison of solubility (solid lines) and permeance (solid points with dotted lines) of pure and mixed CO_2 (blue) and CH_4 (green) for the mixture of (**a**) CO_2 (50 vol.%) / CH_4 (50 vol.%) and (**b**) CO_2 (40 vol.%) / CH_4 (60 vol.%). Solubility calculated using the DMS model with minimized the concentration squared differences. 6

Fig. S7. Comparison of pure and mixed diffusivity at 295 K for CO_2 (blue colour) and CH_4 (green colour) in the case of the DMS model with minimized the concentration squared differences............ 7

Fig. S8. Comparison of total (green), solubility (red) and diffusivity (blue) CO_2 vs. CH_4 selectivity, pure (solid lines) and mixed (dashed lines), for the mixture of (**a**) CO_2 (50 vol.%) / CH_4 (50 vol.%) and (**b**) CO_2 (40 vol.%) / CH_4 (60 vol.%). The case of the DMS model with minimized the concentration squared differences. 7

Fig. S9. Diffusivity of (**a**) pure and (**b**) mixed CO_2 in the polysulfone-based membrane from Air Products' PRISM PA1020-P1 module at 295 K according to the linearized partial immobilization model. A straight dotted line is from the fit. The case of the DMS model with minimized the solubility squared differences. 7

Fig. S10. Diffusivity of (**a**) pure and (**b**) mixed CH_4 in the polysulfone-based membrane from Air Products' PRISM PA1020-P1 module at 295 K according to the linearized partial immobilization model. A straight dotted line is from the fit. The case of the DMS model with minimized the solubility squared differences. 8

Fig. S11. Diffusivity of (**a**) pure and (**b**) mixed CO_2 in the polysulfone-based membrane from Air Products' PRISM PA1020-P1 module at 295 K according to the linearized partial immobilization model. A straight dotted line is from the fit. The case of the DMS model with minimized the concentration squared differences. 8

Fig. S12. Diffusivity of (**a**) pure and (**b**) mixed CH_4 in the polysulfone-based membrane from Air Products' PRISM PA1020-P1 module at 295 K according to the linearized partial immobilization model. A straight dotted line is from the fit. The case of the DMS model with minimized the concentration squared differences. 8

Table S1. Summary of the permeances of CO_2 and CH_4, pure[1] and in the mixture[2] of 50% CO_2 / 50% CH_4, at 295 K in the polysulfone-based membrane from the Air Products' PRISM PA1020-P1 module.

**Pressure (p)[3]**			**Permeance (Q)[4]**				**Q_{mix}/Q_{pure}**		**Selectivity (α)[5]**	
**total**	**partial**		**CO_2**		**CH_4**					
**bar**	**CO_2**	**CH_4**	**pure**	**mix**	**pure**	**mix**	**CO_2**	**CH_4**	**pure**	**mix**
2.3	1.1	1.1	$1.08\cdot10^{-2}$	$6.00\cdot10^{-3}$	$3.15\cdot10^{-4}$	$2.92\cdot10^{-4}$	0.56	0.93	34.2	20.6
3.2	1.6	1.6	$1.08\cdot10^{-2}$	$6.54\cdot10^{-3}$	$3.15\cdot10^{-4}$	$2.81\cdot10^{-4}$	0.61	0.89	34.2	23.3
4.4	2.2	2.2	$1.08\cdot10^{-2}$	$6.47\cdot10^{-3}$	$3.15\cdot10^{-4}$	$2.89\cdot10^{-4}$	0.60	0.92	34.2	22.4
5.8	2.9	2.9	$1.08\cdot10^{-2}$	$5.82\cdot10^{-3}$	$3.15\cdot10^{-4}$	$2.84\cdot10^{-4}$	0.54	0.90	34.2	20.5
7.2	3.6	3.6	$1.08\cdot10^{-2}$	$5.08\cdot10^{-3}$	$3.15\cdot10^{-4}$	$2.78\cdot10^{-4}$	0.47	0.88	34.2	18.2

[1] Janusz-Cygan, A.; Jaschik, J.; Wojdyła, A.; Tańczyk, M. The Separative Performance of Modules with Polymeric Membranes for a Hybrid Adsorptive/Membrane Process of CO_2 Capture from Flue Gas. *Membranes* **2020**, *10*, 309, doi:10.3390/membranes10110309

[2] Janusz-Cygan, A.; Jaschik, J.; Tańczyk, M. Upgrading Biogas from Small Agricultural Sources into Biomethane by Membrane Separation. *Membranes* **2021**, *11*, 938, doi:10.3390/membranes11120938.

[3] p is in bar

[4] Q is in cm^3(STP) cm^{-2} s^{-1} bar^{-1}

[5] α is the ratio of Q_{CO2} and Q_{CH4}

Table S2. Summary of the solubility, diffusivity and selectivity of CO_2 and CH_4, pure and in the mixture of 50% CO_2 / 50% CH_4, at 295 K in the polysulfone-based membrane from the Air Products' PRISM PA1020-P1 module.

**Pressure (p)[1]**			**Solubility (S)[2]**				**Diffusivity (D)[3]**				**S_{mix}/S_{pure}**		**Solubility selectivity (α_S)[4]**		**Diffusivity selectivity (α_D)[5]**	
**total**	**partial**		**CO_2**		**CH_4**		**CO_2**		**CH_4**							
**bar**	**CO_2**	**CH_4**	**pure**	**mix**	**pure**	**mix**	**pure**	**mix**	**pure**	**mix**	**CO_2**	**CH_4**	**pure**	**mix**	**pure**	**mix**
2.3	1.1	1.1	6.00	5.51	1.33	0.84	$1.80\cdot10^{-7}$	$1.09\cdot10^{-7}$	$2.37\cdot10^{-8}$	$3.46\cdot10^{-8}$	0.92	0.64	4.5	6.5	7.6	3.1
3.2	1.6	1.6	5.23	4.80	1.24	0.76	$2.06\cdot10^{-7}$	$1.36\cdot10^{-7}$	$2.54\cdot10^{-8}$	$3.72\cdot10^{-8}$	0.92	0.61	4.2	6.3	8.1	3.7
4.4	2.2	2.2	4.57	4.20	1.15	0.68	$2.36\cdot10^{-7}$	$1.54\cdot10^{-7}$	$2.75\cdot10^{-8}$	$4.23\cdot10^{-8}$	0.92	0.60	4.0	6.2	8.6	3.6
5.8	2.9	2.9	4.10	3.78	1.06	0.63	$2.63\cdot10^{-7}$	$1.54\cdot10^{-7}$	$2.96\cdot10^{-8}$	$4.50\cdot10^{-8}$	0.92	0.59	3.9	6.0	8.9	3.4
7.2	3.6	3.6	3.78	3.50	1.00	0.60	$2.85\cdot10^{-7}$	$1.45\cdot10^{-7}$	$3.15\cdot10^{-8}$	$4.67\cdot10^{-8}$	0.93	0.60	3.8	5.9	9.0	3.1

[1] p is in bar

[2] S is in cm^3(STP) cm^{-3}(membrane) bar^{-1}, calculated at 295 K from the Dual Mode Sorption (DMS) model with minimized the solubility squared differences

[3] D is in cm^2 s^{-1}, calculated at 295 K using the appropriate permeance from Table S1 and the solubility, assuming the membrane active layer thickness of 1 μm

[4] α_S is the ratio of S_{CO2} and S_{CH4}

[5] α_D is the ratio of D_{CO2} and D_{CH4}

Table S3. Summary of the permeances of CO_2 and CH_4, pure[1] and in the mixture[2] of 40% CO_2 / 60% CH_4, at 295 K in the polysulfone-based membrane from the Air Products' PRISM PA1020-P1 module.

Pressure (p)[3]			Permeance (Q)[4]				Q_{mix}/Q_{pure}		Selectivity (α)[5]	
total	partial		CO_2		CH_4					
bar	CO_2	CH_4	pure	mix	pure	mix	CO_2	CH_4	pure	mix
2.3	0.9	1.4	$1.08 \cdot 10^{-2}$	$4.67 \cdot 10^{-3}$	$3.15 \cdot 10^{-4}$	$2.71 \cdot 10^{-4}$	0.43	0.86	34.2	17.2
3.2	1.3	1.9	$1.08 \cdot 10^{-2}$	$6.06 \cdot 10^{-3}$	$3.15 \cdot 10^{-4}$	$2.82 \cdot 10^{-4}$	0.56	0.89	34.2	21.5
4.4	1.8	2.6	$1.08 \cdot 10^{-2}$	$6.24 \cdot 10^{-3}$	$3.15 \cdot 10^{-4}$	$2.81 \cdot 10^{-4}$	0.58	0.89	34.2	22.2
5.8	2.3	3.5	$1.08 \cdot 10^{-2}$	$5.67 \cdot 10^{-3}$	$3.15 \cdot 10^{-4}$	$2.93 \cdot 10^{-4}$	0.53	0.93	34.2	19.4
7.2	2.9	4.3	$1.08 \cdot 10^{-2}$	$4.98 \cdot 10^{-3}$	$3.15 \cdot 10^{-4}$	$2.76 \cdot 10^{-4}$	0.46	0.87	34.2	18.1

[1] Janusz-Cygan, A.; Jaschik, J.; Wojdyła, A.; Tańczyk, M. The Separative Performance of Modules with Polymeric Membranes for a Hybrid Adsorptive/Membrane Process of CO_2 Capture from Flue Gas. *Membranes* **2020**, *10*, 309, doi:10.3390/membranes10110309

[2] Janusz-Cygan, A.; Jaschik, J.; Tańczyk, M. Upgrading Biogas from Small Agricultural Sources into Biomethane by Membrane Separation. *Membranes* **2021**, *11*, 938, doi:10.3390/membranes11120938.

[3] p is in bar

[4] Q is in cm^3(STP) cm^{-2} s^{-1} bar^{-1}

[5] α is the ratio of Q_{CO2} and Q_{CH4}

Table S4. Summary of the solubility, diffusivity and selectivity of CO_2 and CH_4, pure and in the mixture of 40% CO_2 / 60% CH_4, at 295 K in the polysulfone-based membrane from the Air Products' PRISM PA1020-P1 module.

Pressure (p)[1]			Solubility (S)[2]				Diffusivity (D)[3]				S_{mix}/S_{pure}		Solubility selectivity (α_S)[4]		Diffusivity selectivity (α_D)[5]	
total	partial		CO_2		CH_4		CO_2		CH_4							
bar	CO_2	CH_4	pure	mix	pure	mix	pure	mix	pure	mix	CO_2	CH_4	pure	mix	pure	mix
2.3	0.9	1.4	6.48	5.76	1.27	0.87	$1.66 \cdot 10^{-7}$	$0.81 \cdot 10^{-7}$	$2.47 \cdot 10^{-8}$	$3.10 \cdot 10^{-8}$	0.89	0.69	5.1	6.6	6.7	2.6
3.2	1.3	1.9	5.74	5.07	1.19	0.79	$1.88 \cdot 10^{-7}$	$1.19 \cdot 10^{-7}$	$2.65 \cdot 10^{-8}$	$3.57 \cdot 10^{-8}$	0.88	0.66	4.8	6.4	7.1	3.3
4.4	1.8	2.6	5.03	4.44	1.09	0.71	$2.14 \cdot 10^{-7}$	$1.40 \cdot 10^{-7}$	$2.88 \cdot 10^{-8}$	$3.95 \cdot 10^{-8}$	0.88	0.65	4.6	6.2	7.4	3.6
5.8	2.3	3.5	4.48	3.97	1.01	0.65	$2.41 \cdot 10^{-7}$	$1.43 \cdot 10^{-7}$	$3.13 \cdot 10^{-8}$	$4.47 \cdot 10^{-8}$	0.89	0.65	4.4	6.1	7.7	3.2
7.2	2.9	4.3	4.12	3.67	0.94	0.62	$2.62 \cdot 10^{-7}$	$1.36 \cdot 10^{-7}$	$3.34 \cdot 10^{-8}$	$4.46 \cdot 10^{-8}$	0.89	0.65	4.4	5.9	7.8	3.0

[1] p is in bar

[2] S is in cm^3(STP) cm^{-3}(membrane) bar^{-1}, calculated at 295 K from the Dual Mode Sorption (DMS) model with minimized the solubility squared differences

[3] D is in $cm^2 \cdot s^{-1}$, calculated at 295 K using the appropriate permeance from Table S1 and the solubility, assuming the membrane active layer thickness of 1 μm

[4] α_S is the ratio of S_{CO2} and S_{CH4}

[5] α_D is the ratio of D_{CO2} and D_{CH4}

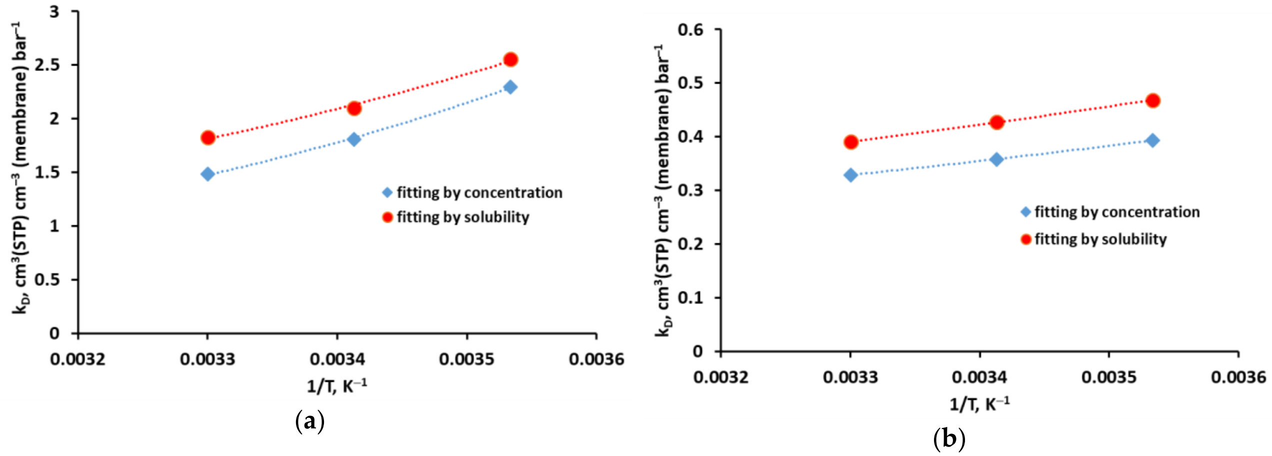

(a) (b)

Figure S1. Temperature dependence of the Henry's constant in the Dual Mode Sorption (DMS) model for (a) CO_2 and (b) CH_4. The dotted lines are from the fit.

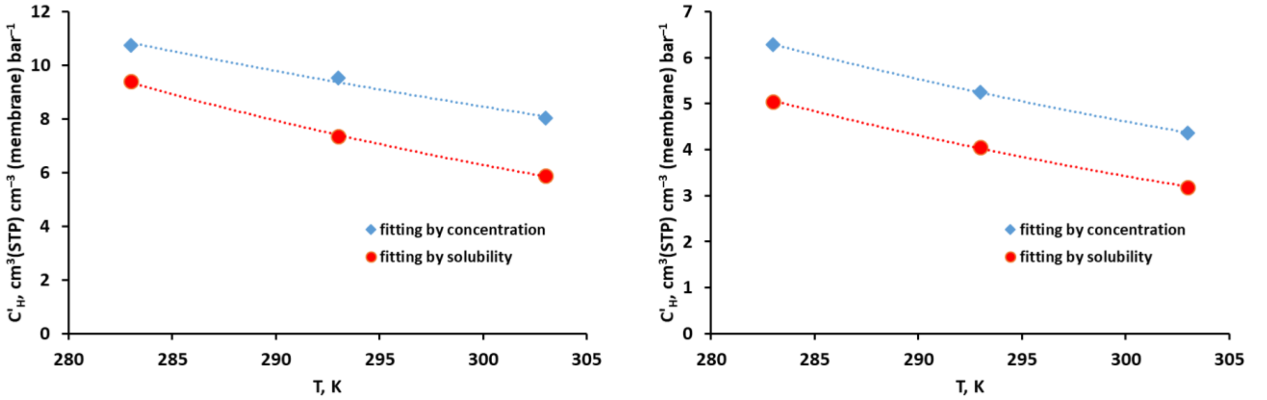

(a) (b)

Figure S2. Temperature dependence of the Langmuir adsorption capacity in the Dual Mode Sorption (DMS) model for (a) CO_2 and (b) CH_4. The dotted lines are from the fit.

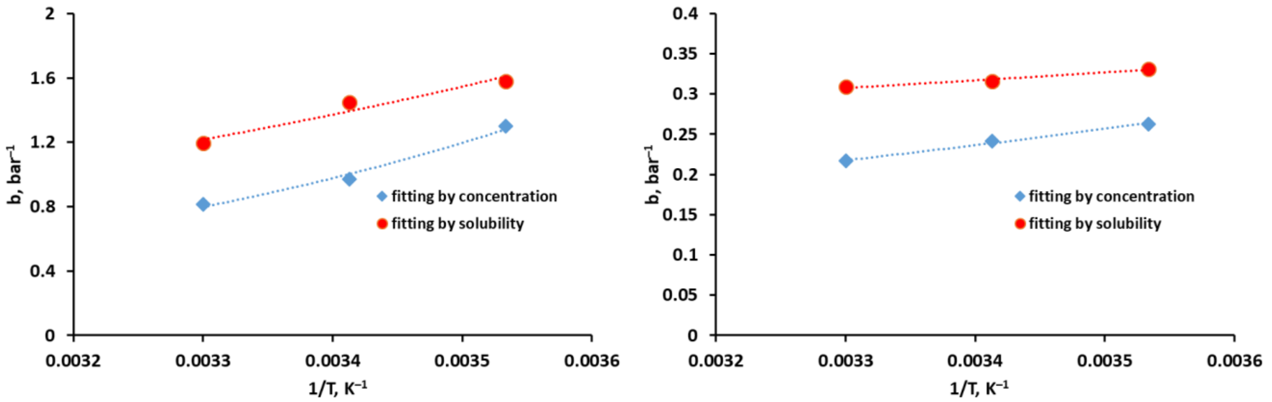

(a) (b)

Figure S3. Temperature dependence of the Langmuir affinity constant in the Dual Mode Sorption (DMS) model for (a) CO_2 and (b) CH_4. The dotted lines are from the fit.

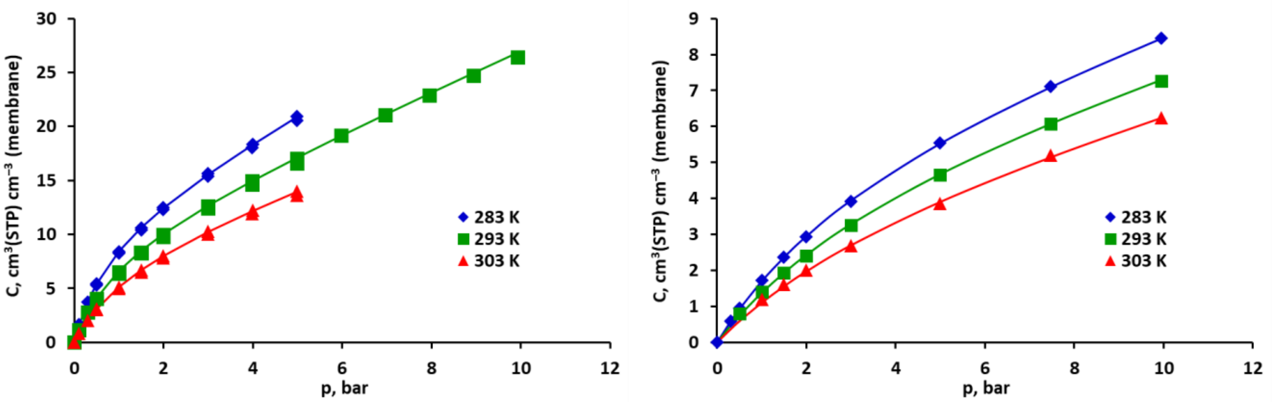

(a) (b)

Figure S4. Concentration of pure (**a**) CO_2 and (**b**) CH_4 in the polysulfone-based membrane from the Air Products' PRISM PA1020-P1 module. Points represent experimental data and lines Dual Mode Sorption (DMS) model predictions (for minimized the concentration squared differences).

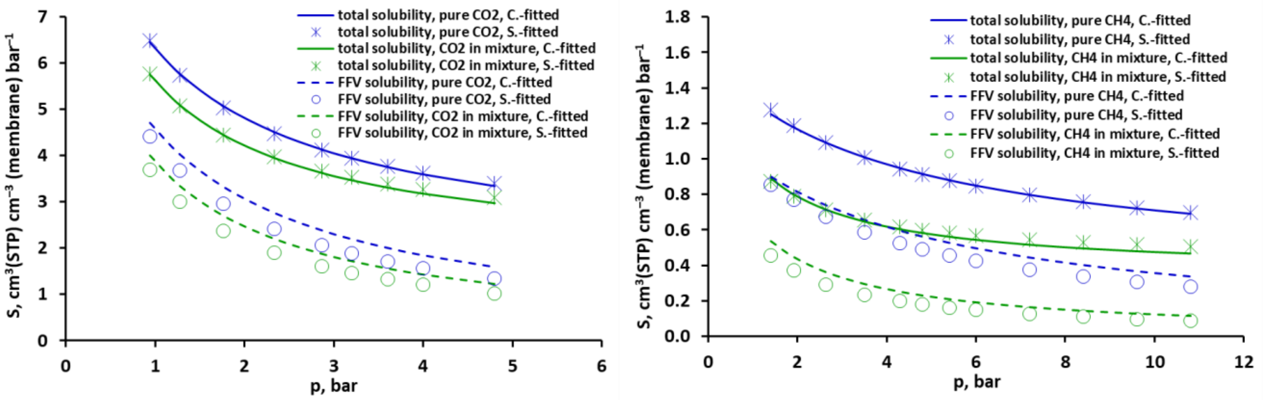

(a) (b)

Figure S5. Total and FFV solubility of (**a**) CO_2 and (**b**) CH_4, pure and mixed (CO_2: 40 vol.% / CH_4: 60 vol.%) at 295 K according to the Dual Mode Sorption (DMS) model predictions for minimized the concentration (blue) and solubility (green) squared differences.

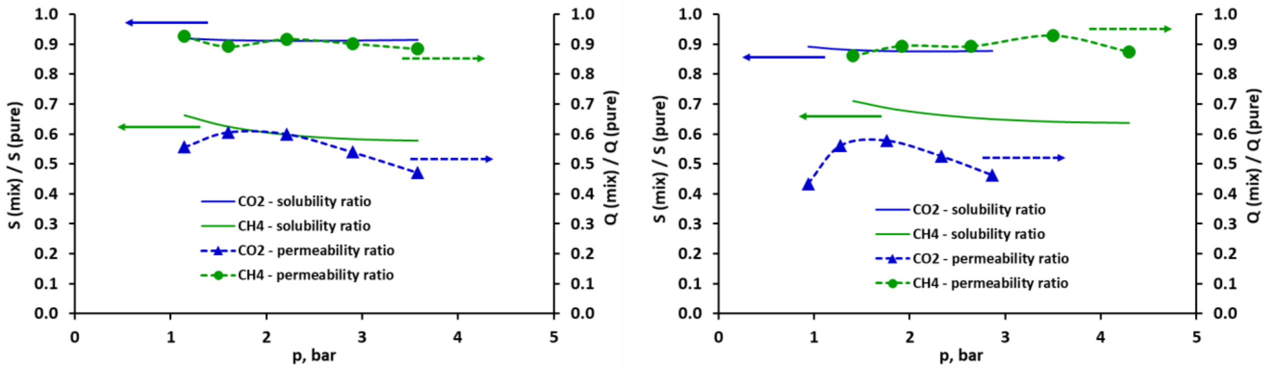

(a) (b)

Figure S6. Comparison of solubility (solid lines) and permeance (solid points with dotted lines) of pure and mixed CO_2 (blue) and CH_4 (green) for the mixture of (**a**) CO_2 (50 vol.%) / CH_4 (50 vol.%) and (**b**) CO_2 (40 vol.%) / CH_4 (60 vol.%). Solubility calculated using the DMS model with minimized the concentration squared differences.

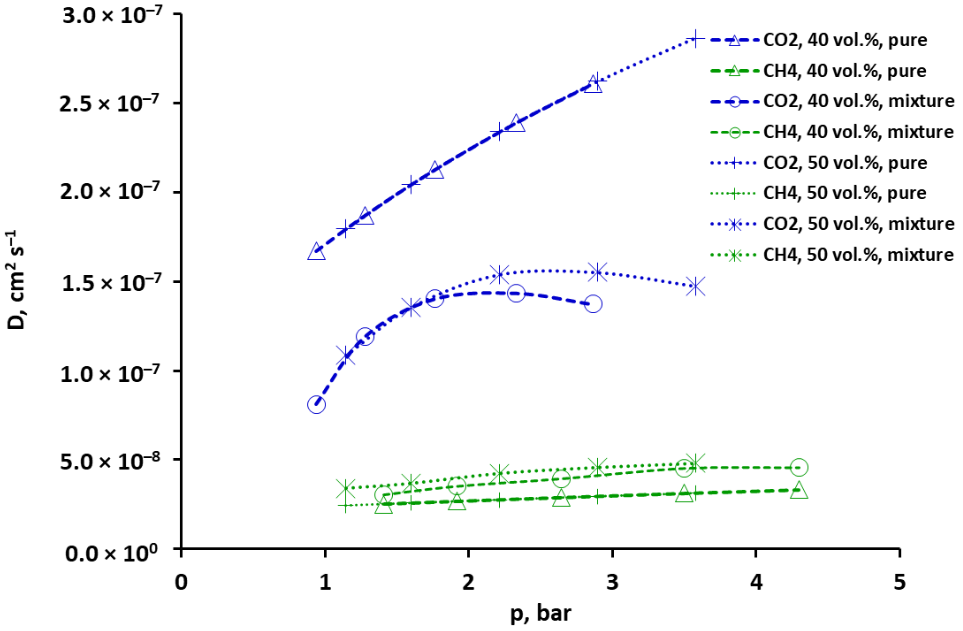

Figure S7. Comparison of pure and mixed diffusivity at 295 K for CO_2 (blue color) and CH_4 (green color) in the case of the DMS model with minimized the concentration squared differences.

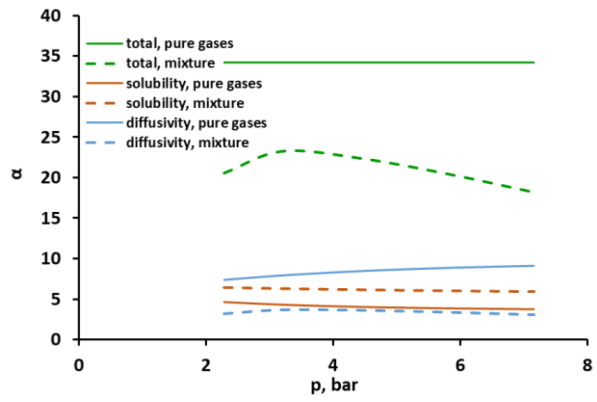

(**a**)

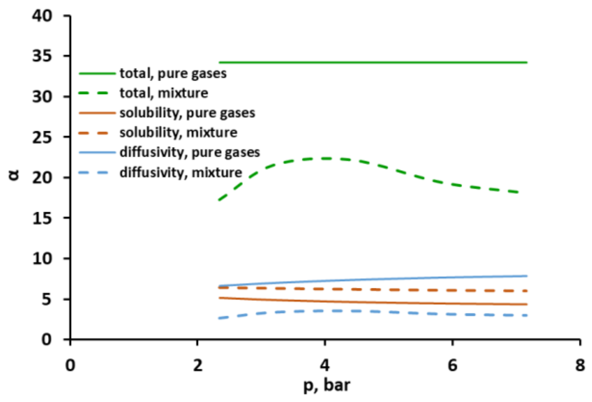

(**b**)

Figure S8. Comparison of total (green), solubility (red) and diffusivity (blue) CO_2 vs. CH_4 selectivity, pure (solid lines) and mixed (dashed lines), for the mixture of (**a**) CO_2 (50 vol.%) / CH_4 (50 vol.%) and (**b**) CO_2 (40 vol.%) / CH_4 (60 vol.%). The case of the DMS model with minimized the concentration squared differences.

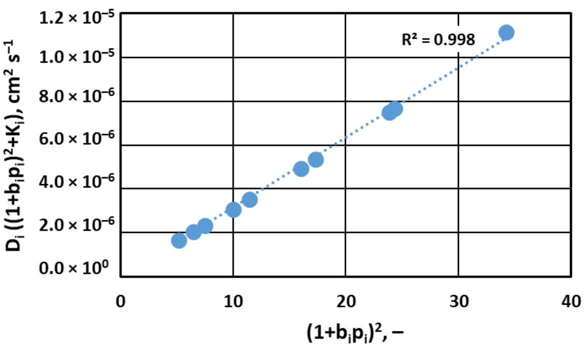

(**a**)

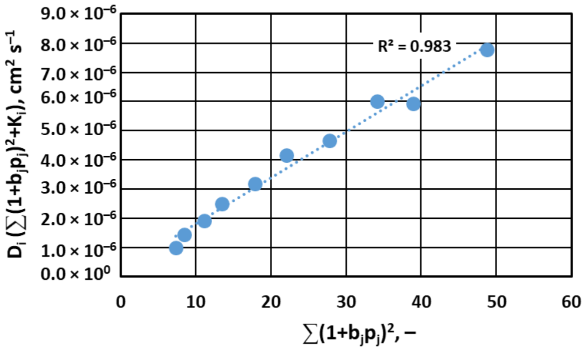

(**b**)

Figure S9. Diffusivity of (**a**) pure and (**b**) mixed CO_2 in the polysulfone-based membrane from Air Products' PRISM PA1020-P1 module at 295 K according to the linearized partial immobilization model. A straight dotted line is from the fit. The case of the DMS model with minimized the solubility squared differences.

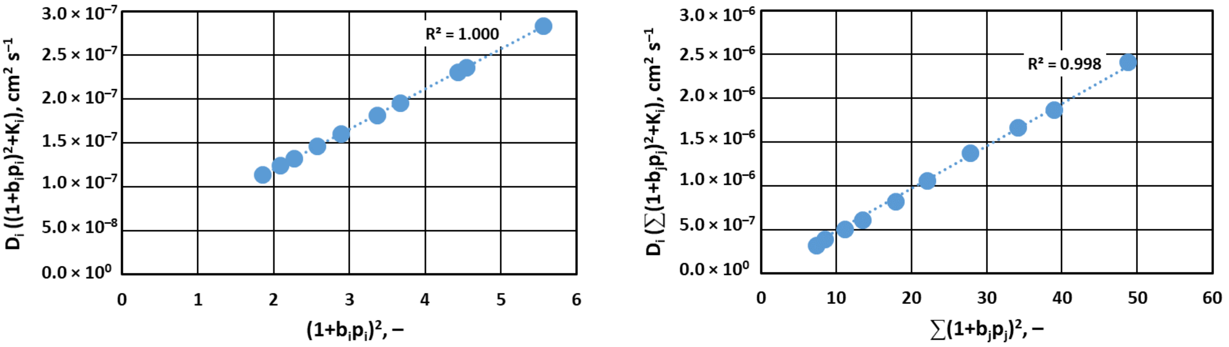

(**a**) (**b**)

Figure S10. Diffusivity of (**a**) pure and (**b**) mixed CH_4 in the polysulfone-based membrane from Air Products' PRISM PA1020-P1 module at 295 K according to the linearized partial immobilization model. The straight dotted lines are from the fit. The case of the DMS model with minimized the solubility squared differences.

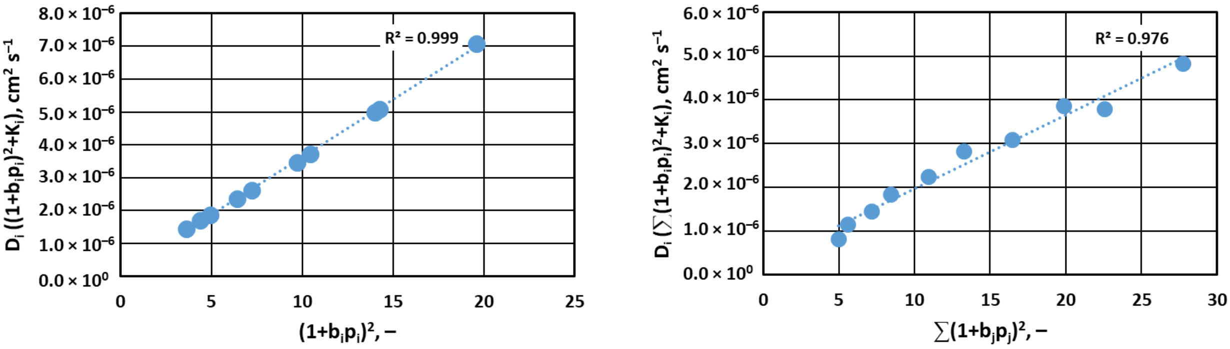

(**a**) (**b**)

Figure S11. Diffusivity of (**a**) pure and (**b**) mixed CO_2 in the polysulfone-based membrane from Air Products' PRISM PA1020-P1 module at 295 K according to the linearized partial immobilization model. The straight dotted lines are from the fit. The case of the DMS model with minimized the concentration squared differences.

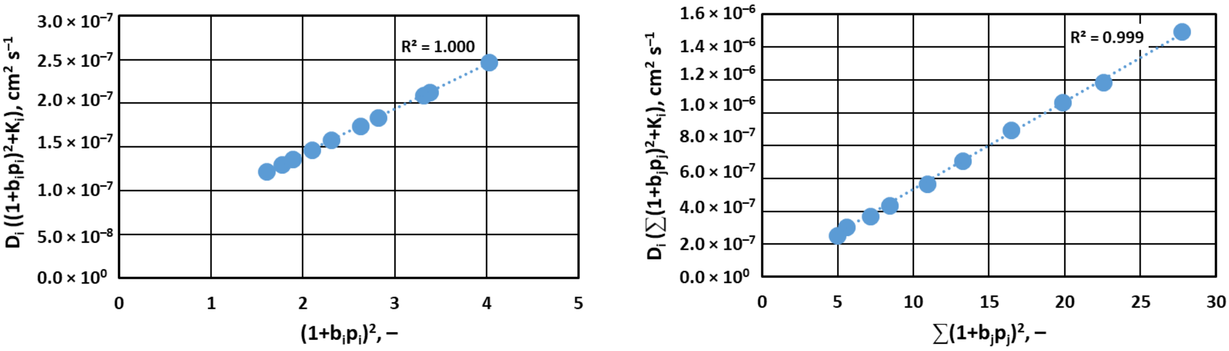

(**a**) (**b**)

Figure S12. Diffusivity of (**a**) pure and (**b**) mixed CH_4 in the polysulfone-based membrane from Air Products' PRISM PA1020-P1 module at 295 K according to the linearized partial immobilization model The straight dotted lines are from the fit. The case of the DMS model with minimized the concentration squared differences.
